# Supplementary material for: Lupus autoantibodies initiate a maladaptive equilibrium sustained by HMGB1:RAGE signaling and reversed by LAIR-1:C1q signaling
Source: Res Sq. 2023 May 22:rs.3.rs-2870168. Preprint. [Version 1] doi: 10.21203/rs.3.rs-2870168/v1 (PMC10246276; doi:10.21203/rs.3.rs-2870168/v1)
Supplement: 1 [file NIHPPRS2870168V1-supplement-1.pdf]

Supplementary Figure 1. Pathology is sustained for at least 12 months. A) Decreased dendritic complexity in 12 month old DNRAb$^+$ compared with DNRAb$^-$ mice (mean +/- SEM; n=4-5 mice per group; n=55-59 neurons analyzed per group; linear mixed model test). **B)** Decreased dendritic spine density in 12 m.o. DNRAb$^+$ compared with DNRAb$^-$ mice (median (solid line) with quartiles (dash); n=4 mice per group; n=15-18 neurons analyzed per group; Mann-Whitney test). **C)** Representative sections of microglia in CA1 stratum radiatum stained for Iba1 (red) and CD68 (white) in 12 m.o. DNRAb$^+$ and DNRAb$^-$ B6.H2^d mice (n=3 mice per group). **D)** Increased activation score in 12 m.o. DNRAb$^+$ microglia compared to 12 m.o. DNRAb$^-$ counterparts based on morphology and CD68 expression (median (solid line) with quartiles (dash); n=3 mice per group; n=110-169 microglia scored per group; Mann-Whitney test).

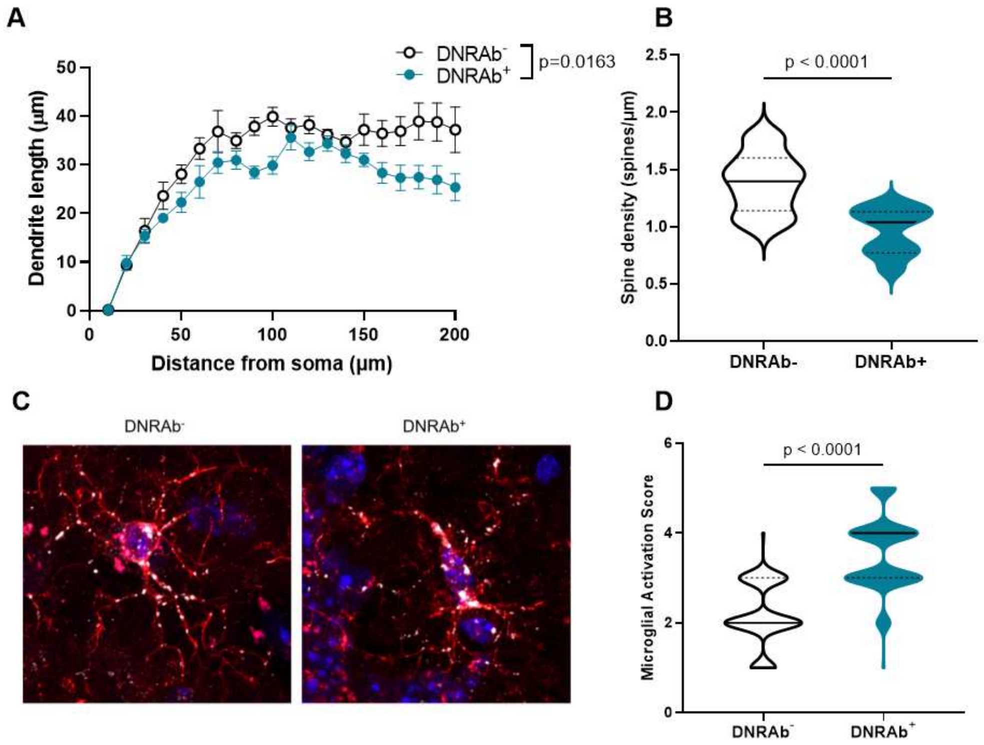

Supplementary Figure 2. Acute neuronal loss is not affected by loss of RAGE or microglial LAIR-1. A) Decreased CA1 neurons in WT (B6) and RAGE KO mDNRAb$^+$ mice compared to their mDNRAb$^-$ counterparts (median (solid line) with quartiles (dash); n=3-4 mice per group; n= 72-97 sections per group; Kruskal-Wallis test). B) Decreased CA1 neurons in LAIR-1 cKO DNRAb$^+$ mice compared to DNRAb$^-$ (median (solid line) with quartiles (dash); n=3 mice per group; n= 67-98 sections per group; Mann-Whitney test).

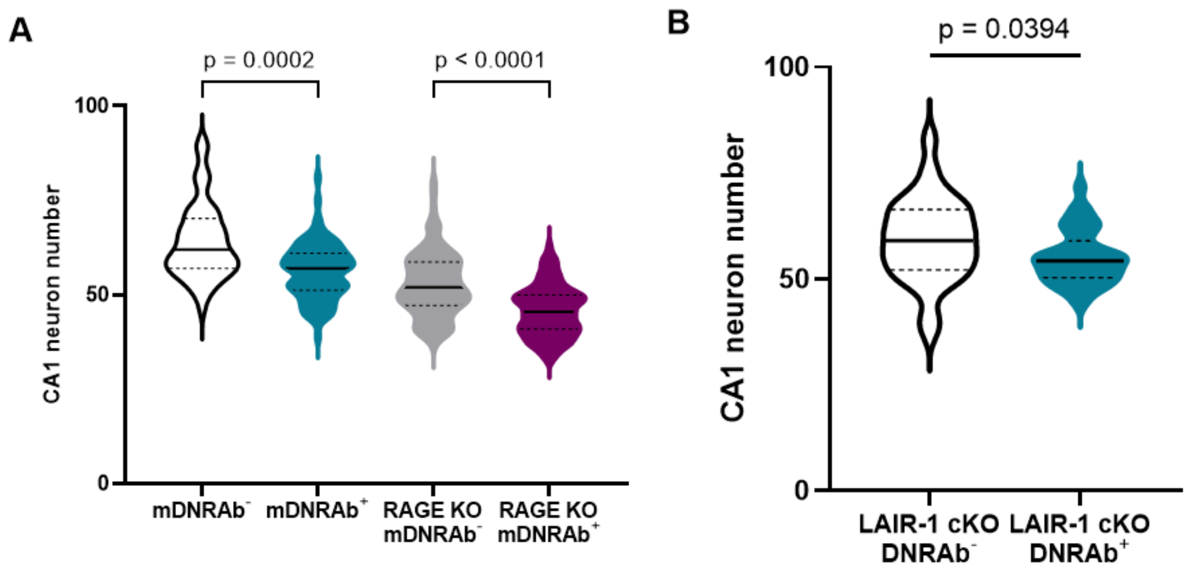

Supplementary Figure 3. Single-cell RNA-seq clustering and quality control. A) UMAP plot colored by clustering. **B)** UMAP plot split by mouse of origin, colored by cell type. No evidence of strong batch effects in the UMAP space. **C)** Violin plot of various QC metrics of interest, with similar distributions observed in each mouse. QC metrics include the score returned by Azimuth (Azimuth Score), number of genes per cell (nGene), number of UMI per cell (nUMI), percent UMI coming from mitochondrial reads (Percent Mitochondrial), percent UMI mapping to ribosomal proteins (Percent Ribosomal Protein), and doublet scores (scds). **D)** Feature plots of genes associated with a known microglia activation signature[48]. Subclustering within microglia subtypes is largely driven by these variables. **E)** Feature plots of number of genes per cell (nGene) and number of UMIs per cell (nUMI). Subclustering within microglia subtypes is largely driven by these variables.

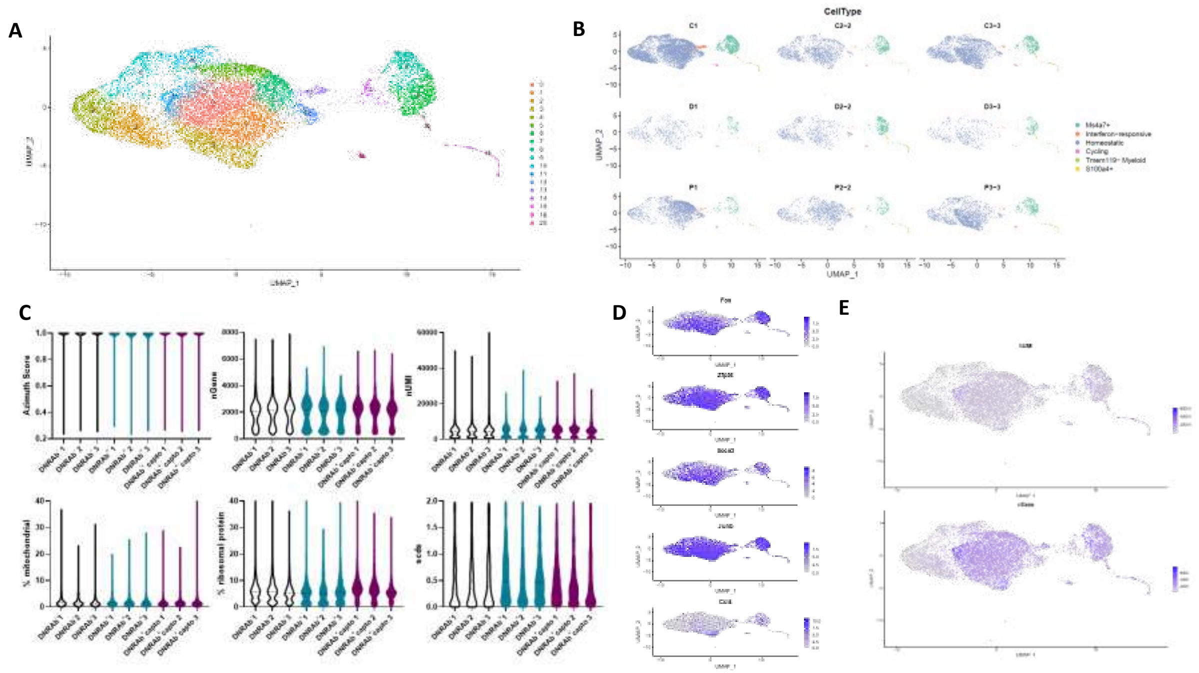

Supplementary Figure 4. Concordance score of Ms4a7+ microglia with known microglial gene signatures. A) Higher DAM signature gene set score in Ms4a7+ compared with Homeostatic microglia (Keren-Shaul et al. (2017) [26]; median (solid line) with quartiles (dash); n=3 mice per group; n=2515-15285 cells/cluster; Mann-Whitney test). **B)** Lower Homeostatic signature gene set score in Homeostatic compared with Ms4a7+ cluster (Keren-Shaul et al. (2017) [26]; median (solid line) with quartiles (dash); n=3 mice per group; n=2515-15285 cells/cluster; Mann-Whitney test). **C)** Higher NPSLE signature gene set score in Ms4a7+ compared with Homeostatic microglia (Makinde et al. (2020) [29]; median (solid line) with quartiles (dash); n=3 mice per group; n=2515-15285 cells/cluster; Mann-Whitney test). **D)** Higher MGnD signature gene set score in Ms4a7+ compared with Homeostatic microglia (Krasemann et al. (2017) [30]; median (solid line) with quartiles (dash); n=3 mice per group; n=2515-15285 cells/cluster; Mann-Whitney test).

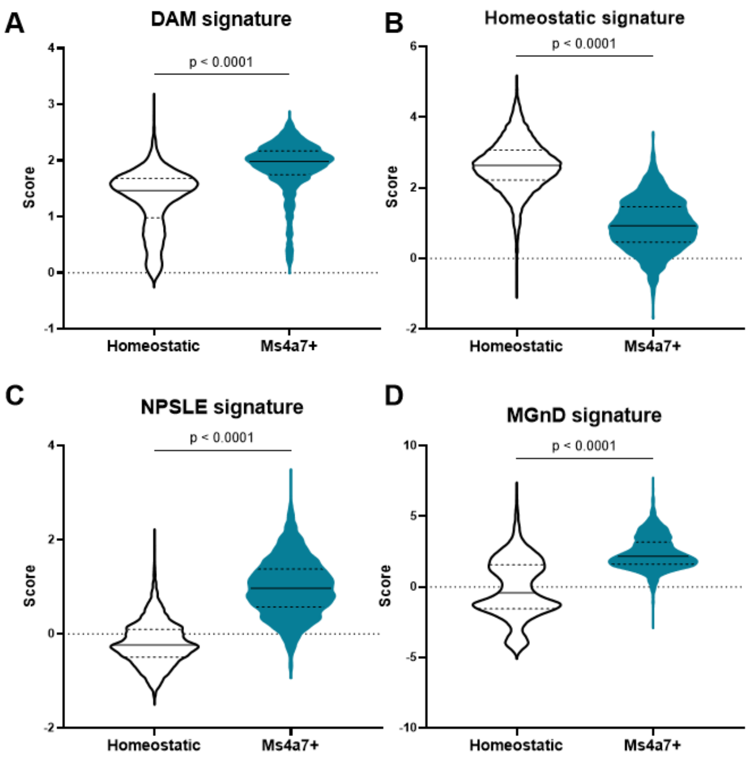

Supplementary Table 1. Single-cell RNA-seq quality control metrics summary. For each sample (n=3 mice per treatment group), estimated number of cells; mean reads per cell; median genes per cell; number of reads; valid barcodes; sequencing saturation; Q30 bases in barcode, RNA read, and UMI; reads mapped to genome; reads mapped confidently to genome, intergenic regions, intronic regions, exonic regions, and transcriptome; reads mapped to antisense to gene; fraction of reads in cells; total genes detected; and median UMI counts per cell.

	DNRAb$^-$ 1	DNRAb$^-$ 2	DNRAb$^-$ 3	DNRAb$^+$ 1	DNRAb$^+$ 2	DNRAb$^+$ 3	DNRAb$^+$ capto 1	DNRAb$^+$ capto 2	DNRAb$^+$ capto 3
Estimated Number of Cells	6119	1868	3092	763	1257	754	2189	1445	2306
Mean Reads per Cell	24574	77412	45456	205512	130434	194448	71837	108980	66490
Median Genes per Cell	2025	2778	2504	2738	2664	2986	2815	2889	2716
Number of Reads	150370974	144605605	140548918	156805849	163955607	146613789	157251130	157476807	153326132
Valid Barcodes	0.977	0.976	0.976	0.975	0.973	0.974	0.977	0.975	0.975
Sequencing Saturation	0.48	0.707	0.595	0.874	0.8	0.86	0.73	0.791	0.696
Q30 Bases in Barcode	0.978	0.978	0.976	0.978	0.978	0.978	0.978	0.976	0.976
Q30.Bases in RNA Read	0.883	0.891	0.882	0.894	0.89	0.887	0.897	0.89	0.88
Q30 Bases in UMI	0.978	0.978	0.974	0.978	0.978	0.978	0.978	0.975	0.974
Reads Mapped to Genome	0.945	0.926	0.939	0.923	0.909	0.921	0.948	0.933	0.936
Reads Mapped Confidently to Genome	0.93	0.91	0.924	0.908	0.893	0.905	0.932	0.917	0.92
Reads Mapped Confidently to Intergenic Regions	0.042	0.044	0.043	0.046	0.047	0.043	0.043	0.042	0.04
Reads Mapped Confidently to Intronic Regions	0.365	0.365	0.378	0.382	0.379	0.384	0.387	0.395	0.392
Reads Mapped Confidently to Exonic Regions	0.522	0.5	0.504	0.48	0.467	0.479	0.503	0.48	0.488
Reads Mapped Confidently to Transcriptome	0.476	0.454	0.457	0.434	0.421	0.433	0.459	0.436	0.446
Reads Mapped Antisense to Gene	0.026	0.027	0.027	0.027	0.026	0.027	0.024	0.026	0.024
Fraction Reads in Cells	0.801	0.773	0.79	0.764	0.77	0.784	0.865	0.847	0.85
Total Genes Detected	18639	17196	17670	15776	17136	16388	17051	17162	17638
Median UMI Counts per Cell	4481	7153	5927	7186	7322	8300	7266	7805	6570

Supplementary Table 2. Single-cell RNA-seq cell cluster composition frequency and percentage. For each sample (n=3 mice per treatment group), number of cells in each cluster and the percentage of total cells this represents.

	DNRAb^{-} 1	DNRAb^{-} 2	DNRAb^{-} 3	DNRAb^{+} 1	DNRAb^{+} 2	DNRAb^{+} 3	DNRAb^{+} capto 1	DNRAb^{+} capto 2	DNRAb^{+} capto 3
Number of cells in Homeostatic cluster	4924	1431	2476	537	709	457	1848	1119	1784
% of total cells	83.13355	80.89316	84.01765	80.63063	64.396	68.92911	89.57828	86.20955	83.40346
Number of cells in Ms4a7+ cluster	741	294	378	93	305	157	131	123	293
% of total cells	12.51055	16.61956	12.8266	13.96396	27.70209	23.68024	6.349976	9.476117	13.69799
Number of cells in Tmem119- cluster	41	17	38	15	50	18	23	33	34
% of total cells	0.692217	0.960995	1.289447	2.252252	4.541326	2.714932	1.114881	2.542373	1.589528
Number of cells in IFN-responsive cluster	163	15	29	6	7	6	39	15	16
% of total cells	2.751984	0.847937	0.984052	0.900901	0.635786	0.904977	1.890451	1.155624	0.748013
Number of cells in Cycling cluster	44	9	17	10	9	4	18	5	9
% of total cells	0.742867	0.508762	0.576858	1.501502	0.817439	0.603318	0.872516	0.385208	0.420757
Number of cells in S100a4+ cluster	10	3	9	5	21	21	4	3	3
% of total cells	0.168833	0.169587	0.305395	0.750751	1.907357	3.167421	0.193892	0.231125	0.140252

Supplementary Table 3. Single-cell RNA-seq cell cluster comparison statistics. For each cluster, ANOVA p-value, FDR adjusted p-value, means of the groups compared, and F statistic are reported (n=3 mice per treatment group). Posthoc pairwise t tests were performed between each treatment group within each cluster; comparisons performed, pairwise p values, and Holm-Sidak adjusted p values are reported (n=3 mice per treatment group).

Cluster	Treatment	ANOVA p-value	FDR adjusted p-value	Mean	F statistic	Comparison	Pairwise p-value	Adjusted p-value
Homeostatic	DNRAb$^-$	0.004518	0.01477	0.82681	9.919631	DNRAb$^+$ vs DNRAb$^-$	0.0117396	0.034807
	DNRAb$^+$			0.71319		DNRAb$^+$ vs DNRAb$^+$ capto	0.0016230	0.004861
	DNRAb$^+$ captopril			0.86397		DNRAb$^+$ capto vs DNRAb$^-$	0.2494213	0.577148
Ms4a7+	DNRAb$^-$	0.013422	0.026844	0.13986	6.948678	DNRAb$^+$ vs DNRAb$^-$	0.0521964	0.148559
	DNRAb$^+$			0.21782		DNRAb$^+$ vs DNRAb$^+$ capto	0.0042981	0.012839
	DNRAb$^+$ captopril			0.09841		DNRAb$^+$ capto vs DNRAb$^-$	0.1673378	0.422693
Tmem119-	DNRAb$^-$	0.022958	0.034437	0.00981	5.712187	DNRAb$^+$ vs DNRAb$^-$	0.0075268	0.022411
	DNRAb$^+$			0.03169		DNRAb$^+$ vs DNRAb$^+$ capto	0.0770633	0.213831
	DNRAb$^+$ captopril			0.01749		DNRAb$^+$ capto vs DNRAb$^-$	0.1971263	0.482463
IFN-responsive	DNRAb$^-$	0.488584	0.488584	0.01528	0.771867	DNRAb$^+$ vs DNRAb$^-$	0.2552766	0.058696
	DNRAb$^+$			0.00814		DNRAb$^+$ vs DNRAb$^+$ capto	0.4146736	0.799463
	DNRAb$^+$ captopril			0.01265		DNRAb$^+$ capto vs DNRAb$^-$	0.7287750	0.980048
Cycling	DNRAb$^-$	0.466644	0.488584	0.00609	0.82538	DNRAb$^+$ vs DNRAb$^-$	0.3495265	0.724774
	DNRAb$^+$			0.00974		DNRAb$^+$ vs DNRAb$^+$ capto	0.2556850	0.587645
	DNRAb$^+$ captopril			0.00559		DNRAb$^+$ capto vs DNRAb$^-$	0.8266030	0.994787
S100a4+	DNRAb$^-$	0.004923	0.01477	0.00214	9.660058	DNRAb$^+$ vs DNRAb$^-$	0.0039998	0.011952
	DNRAb$^+$			0.01942		DNRAb$^+$ vs DNRAb$^+$ capto	0.0033397	0.009985
	DNRAb$^+$ captopril			0.00188		DNRAb$^+$ capto vs DNRAb$^-$	0.9129096	0.999339

Supplementary Table 4. Single-cell RNA-seq gene expression in Ms4a7+ cluster by sample.

For each sample (n=3 mice per treatment group), transcripts per million (TPM) and log_2TPM for each indicated gene (*Tnf, Il1b, C3ar1, Lyz2, Ifnar1, Cd93, Axl, Lair1*).

Gene	DNRAb$^-$ 1	DNRAb$^-$ 2	DNRAb$^-$ 3	DNRAb$^+$ 1	DNRAb$^+$ 2	DNRAb$^+$ 3	DNRAb$^+$ capto 1	DNRAb$^+$ capto 2	DNRAb$^+$ capto 3
Tnf									
TPM	78.75947	136.7593	119.0401	130.1744	139.7316	162.5265	44.29625	65.39778	154.4324
Log$_2$TPM	6.317584	7.106006	6.907372	7.035343	7.136802	7.353381	5.50132	6.053063	7.280143
Il1b									
TPM	125.6192	285.5573	69.24553	147.6606	269.6664	540.32	18.79235	117.716	233.2621
Log$_2$TPM	6.984352	8.16268	6.134335	7.215878	8.080372	9.080338	4.306871	6.891371	7.87198
C3ar1									
TPM	405.4573	352.4923	369.1798	462.4107	443.4286	433.7629	408.0625	417.2378	420.4249
Log$_2$TPM	8.66696	8.465535	8.532082	8.856147	8.795808	8.764085	8.676177	8.70818	8.719132
Lyz2									
TPM	7777.828	7907.481	7644.24	9634.851	9430.076	10136.92	6151.81	9920.843	7553.358
Log$_2$TPM	12.92534	12.94918	12.90035	13.2342	13.20321	13.30747	12.58703	13.27639	12.88309
Ifnar1									
TPM	152.459	178.6539	150.1617	172.9183	194.9023	213.1143	167.7888	164.8024	109.2552
Log$_2$TPM	7.261709	7.489076	7.239948	7.442266	7.613991	7.742237	7.399076	7.373321	6.784702
Cd93									
TPM	150.039	154.5765	175.837	178.747	199.5429	172.2136	157.0504	187.0376	85.74455
Log$_2$TPM	7.238777	7.281481	7.466276	7.489824	7.647767	7.436408	7.30424	7.554878	6.438701
Axl									
TPM	129.3591	152.1688	141.2142	114.6312	183.5588	220.6486	130.2041	198.8092	123.5459
Log$_2$TPM	7.026348	7.258979	7.151922	6.853387	7.527937	7.792131	7.035669	7.642479	6.960534
Lair1									
TPM	439.7771	422.7982	430.645	415.781	407.8512	389.6332	437.5933	395.0026	441.6305
Log$_2$TPM	8.783905	8.727234	8.753701	8.703146	8.675432	8.609671	8.77674	8.629366	8.789959

Supplementary Table 5. Single-cell RNA-seq Ms4a7+ cell gene expression comparison statistics. For each gene, comparisons between each group were performed using a likelihood ratio test with FDR and Benjamini-Hochberg corrections. LogFC, logCPM, LR, p-value, and adjusted p-value (padj) are reported (n=3 mice per treatment group).

Gene	Comparison	logFC	logCPM	LR	p-value	padj
Tnf	DNRAb$^+$ vs DNRAb$^-$	0.475008	6.977014	6.651073	0.00991	0.151184
	DNRAb$^+$ vs DNRAb$^+$ capto	0.756772	6.935088	5.489881	0.019127	0.32575
	DNRAb$^+$ capto vs DNRAb$^-$	-0.29284	6.696582	0.733314	0.391811	0.993521
Il1b	DNRAb$^+$ vs DNRAb$^-$	1.092901	7.897332	6.787678	0.009179	0.143578
	DNRAb$^+$ vs DNRAb$^+$ capto	1.467294	7.871111	5.907503	0.015077	0.287376
	DNRAb$^+$ capto vs DNRAb$^-$	-0.36953	7.197758	0.343762	0.557666	0.993521
C3ar1	DNRAb$^+$ vs DNRAb$^-$	0.32206	8.688049	7.359287	0.006672	0.116852
	DNRAb$^+$ vs DNRAb$^+$ capto	0.154019	8.78866	0.790979	0.373804	0.892005
	DNRAb$^+$ capto vs DNRAb$^-$	0.179949	8.638248	1.250056	0.263542	0.993521
Lyz2	DNRAb$^+$ vs DNRAb$^-$	0.403402	13.11144	12.55886	0.000394	0.015454
	DNRAb$^+$ vs DNRAb$^+$ capto	0.345297	13.14444	2.232687	0.135119	0.701726
	DNRAb$^+$ capto vs DNRAb$^-$	0.069946	12.95071	0.097732	0.754569	0.998601
Ifnar1	DNRAb$^+$ vs DNRAb$^-$	0.37003	7.470336	7.467909	0.006281	0.111822
	DNRAb$^+$ vs DNRAb$^+$ capto	0.465003	7.461662	4.029725	0.044705	0.475877
	DNRAb$^+$ capto vs DNRAb$^-$	-0.09564	7.275785	0.190183	0.662764	0.993521
Cd93	DNRAb$^+$ vs DNRAb$^-$	0.295406	7.430908	5.597676	0.017984	0.21385
	DNRAb$^+$ vs DNRAb$^+$ capto	0.410382	7.39705	2.13733	0.143752	0.71292
	DNRAb$^+$ capto vs DNRAb$^-$	-0.11804	7.253963	0.183124	0.668702	0.993521
Axl	DNRAb$^+$ vs DNRAb$^-$	0.417811	7.310917	5.867303	0.015425	0.193616
	DNRAb$^+$ vs DNRAb$^+$ capto	0.267126	7.402549	0.919316	0.337655	0.876081
	DNRAb$^+$ capto vs DNRAb$^-$	0.137412	7.193658	0.380254	0.537467	0.993521
Lair1	DNRAb$^+$ vs DNRAb$^-$	-0.01522	8.720331	0.018162	0.892797	0.979846
	DNRAb$^+$ vs DNRAb$^+$ capto	-0.01919	8.731565	0.012642	0.910478	0.991134
	DNRAb$^+$ capto vs DNRAb$^-$	0.013904	8.752519	0.008258	0.927592	0.998601

Extended Figure Legends

Figure 1.

B) Mann-Whitney U=948.5; p<0.0001. Median of WT=4.000, n=65, sum of ranks=5422; median of HMGB1 cKO=3.000, n=65, sum of ranks=3094. Difference between medians: -1.000 (actual); -1.000 (Hodges-Lehmann).

C) *Tnf*: ANOVA F=320.1; DFn=1.373, DFd=4.119; R^2=0.9907; Geisser-Greenhouse's epsilon=0.6865; p<0.0001. Matching efficacy F=3.232; R^2=0.01478; p=0.103. Control mean=0.000, n=4; 500 ng/ml HMGB1 mean=1.822, n=4; 1 µg/ml HMGB1 mean=2.277, n=4. Tukey's multiple comparisons test: Control vs. 500 ng/ml HMGB1; mean difference with 95% CI=-1.822, -2.317 to -1.327; SE=0.1185; DF=3; q=21.74; padj=0.0013. Control vs. 1 µg/ml HMGB1; mean difference with 95% CI=-2.277, -2.688 to -1.866; SE=0.09840; DF=3; q=32.73; padj=0.0004. 500 ng/ml HMGB1 vs. 1 µg/ml HMGB1; mean difference with 95% CI=-0.4553, -0.7020 to -0.2086; SE=0.05903; DF=3; q=10.91; padj=0.0093.

***Il1b*:** ANOVA F=158.4; DFn=1.777, DFd=5.331; R^2=0.9814; Geisser-Greenhouse's epsilon=0.8885; p<0.0001. Matching efficacy F=2.262; R^2=0.02059; p=0.1816. Control mean=0.000, n=4; 500 ng/ml HMGB1 mean=2.085, n=4; 1 µg/ml HMGB1 mean=2.824, n=4. Tukey's multiple comparisons test: Control vs. 500 ng/ml HMGB1; mean difference with 95% CI=-2.085, -2.693 to -1.478; SE=0.1454; DF=3; q=20.28; padj=0.0015. Control vs. 1 µg/ml HMGB1; mean difference with 95% CI=-2.824, -3.624 to -2.025; SE=0.1913; DF=3; q=20.88; padj=0.0014. 500 ng/ml HMGB1 vs. 1 µg/ml HMGB1; mean difference with 95% CI=-0.7390, -1.380 to -0.09776; SE=0.1535; DF=3; q=6.811; padj=0.0344.

***C1qa*:** ANOVA F=10.58; DFn=1.457, DFd=4.372; R^2=0.7791; Geisser-Greenhouse's epsilon=0.7287; p=0.0238. Matching efficacy F=1.289; R^2=0.1246; p=0.3609. Control

mean=0.000, n=4; 500 ng/ml HMGB1 mean=0.2077, n=4; 1 μg/ml HMGB1 mean=0.2965, n=4. Tukey's multiple comparisons test: Control vs. 500 ng/ml HMGB1; mean difference with 95% CI=-0.2077, -0.3810 to -0.03447; SE=0.04146; DF=3; q=7.085; padj=0.0309. Control vs. 1 μg/ml HMGB1; mean difference with 95% CI=-0.2965, -0.6058 to 0.01275; SE=0.07401; DF=3; q=5.666; padj=0.0557. 500 ng/ml HMGB1 vs. 1 μg/ml HMGB1; mean difference with 95% CI=-0.08880, -0.4107 to 0.2331; SE=0.07703; DF=3; q=1.630; padj=0.5515.

D) TNFα: Paired t-test t=21.97, df=4; p<0.0001. Difference mean=4611; SD=469.3; SEM=209.9; 95% CI=4028 to 5194; R^2=0.9918. Pairing r=0.3097; p=0.3060.

IL-1β: Paired t-test t=5.040, df=4; p=0.0073. Difference mean=160.3; SD=71.12; SEM=31.80; 95% CI=71.99 to 248.6; R^2=0.8639. Pairing r = horizontal line.

E) *Ifnb*: ANOVA F=51.33; DFn=1.110, DFd=2.220; R^2=0.9625; Geisser-Greenhouse's epsilon=0.5550; p=0.0141. Matching efficacy F=3.222; R^2=0.05697; p=0.1467. Control mean=0.000, n=3; 100 ng/ml HMGB1 mean=1.279, n=3; 1 μg/ml HMGB1 mean=2.942, n=3. Tukey's multiple comparisons test: Control vs. 100 ng/ml HMGB1; mean difference with 95% CI=-1.279, -3.627 to 1.068; SE=0.3985; DF=2; q=4.541; padj=0.1504. Control vs. 1 μg/ml HMGB1; mean difference with 95% CI=-2.942, -4.416 to -1.468; SE=0.2502; DF=2; q=16.63; padj=0.0130. 100 ng/ml HMGB1 vs. 1 μg/ml HMGB1; mean difference with 95% CI=-1.663, -2.733 to -0.5926; SE=0.1817; DF=2; q=12.94; padj=0.0213.

***Irf7*:** ANOVA F=31.57; DFn=1.343, DFd=4.028; R^2=0.9132; Geisser-Greenhouse's epsilon=0.6713; p=0.0042. Matching efficacy F=3.362; R^2=0.1273; p=0.0962. Control mean=0.000, n=4; 500 ng/ml HMGB1 mean=0.8098, n=4; 1 μg/ml HMGB1 mean=1.130, n=4. Tukey's multiple comparisons test: Control vs. 500 ng/ml HMGB1; mean difference with 95% CI=-0.8098, -1.481 to -0.1389; SE=0.1605; DF=3; q=7.133; padj=0.0304. Control vs. 1 μg/ml

HMGB1; mean difference with 95% CI=-1.130, -1.875 to -0.3845; SE=0.1783; DF=3; q=8.959; padj=0.0162. 500 ng/ml HMGB1 vs. 1 µg/ml HMGB1; mean difference with 95% CI=-0.3198, -0.6658 to 0.02616; SE=0.08280; DF=3; q=5.463; padj=0.0612.

***Mx1*:** ANOVA F=99.88; DFn=1.481, DFd=4.443; R^2=0.9708; Geisser-Greenhouse's epsilon=0.7405; p=0.0002. Matching efficacy F=1.322; R^2=0.01891; p=0.3517. Control mean=0.000, n=4; 500 ng/ml HMGB1 mean=1.505, n=4; 1 µg/ml HMGB1 mean=1.936, n=4. Tukey's multiple comparisons test: Control vs. 500 ng/ml HMGB1; mean difference with 95% CI=-1.505, -2.177 to -0.8331; SE=0.1608; DF=3; q=13.23; padj=0.0053. Control vs. 1 µg/ml HMGB1; mean difference with 95% CI=-1.936, -2.321 to -1.551; SE=0.09212; DF=3; q=29.71; padj=0.0005. 500 ng/ml HMGB1 vs. 1 µg/ml HMGB1; mean difference with 95% CI=-0.4305, -1.126 to 0.2650; SE=0.1664; DF=3; q=3.658; padj=0.1565.

F) Paired t-test t=6.100, df=4; p=0.0037. Difference mean=98.68; SD=36.17; SEM=16.18 95% CI=53.76 to 143.6; R^2=0.9029. Pairing r = horizontal line.

G) ANOVA F=55.05; DFn=1.001, DFd=2.003; R^2=0.9649; Geisser-Greenhouse's epsilon=0.5007; p=0.0176. Matching efficacy F=3.953; R^2=0.06480; p=0.1129. Control mean=0.000, n=3; 10^3 Units IFNβ mean=0.1876, n=3; 10^5 Units IFNβ mean=0.3823, n=3. Tukey's multiple comparisons test: Control vs. 10^3 Units IFNβ; mean difference with 95% CI=-0.1876, -0.4649 to 0.08965; SE=0.04707; DF=2; q=5.637; padj=0.1031. Control vs. 10^5 Units IFNβ; mean difference with 95% CI=-0.3823, -0.6278 to -0.1369; SE=0.04167; DF=2; q=12.98; padj=0.0212. 10^3 Units IFNβ vs. 10^5 Units IFNβ; mean difference with 95% CI=-0.1947, -0.2276 to -0.1618; SE=0.005587; DF=2; q=49.28; padj=0.0004.

Figure 2.

A) ***Tnf***: Two-way repeated measures ANOVA; genotype (WT vs RAGE KO) x HMGB1

concentration (0, 100, 1000 ng/ml)=4.619% of total variation, F=88.24, DFn=2, DFd=8, p<0.0001; HMGB1 concentration=90.96% of total variation, F=1738, DFn=1.321, DFd=5.283, p<0.0001; genotype =3.975% of total variation, F=66.39, DFn=1, DFd=4, p=0.0012; subject (n=3 per genotype; microglia cultured from 3 independent litters per genotype)=0.2395% of total variation, F=2.288, DFn=4, DFd=8, p=0.1484. WT mean=0.9516; RAGE KO mean=0.6168; difference between means=0.3348; SE=0.04109; 95% CI=0.2207 to 0.4489. Geisser-Greenhouse's epsilon=0.6603.

Il1b: Two-way repeated measures ANOVA; genotype (WT vs RAGE KO) x HMGB1 concentration (0, 100, 1000 ng/ml)=3.650% of total variation, F=45.82, DFn=2, DFd=8, p<0.0001; HMGB1 concentration=88.20% of total variation, F=1107, DFn=1.240, DFd=4.961, p<0.0001; genotype =7.293% of total variation, F=54.29, DFn=1, DFd=4, p=0.0018; subject (n=3 per genotype; microglia cultured from 3 independent litters per genotype)=0.5373% of total variation, F=3.373, DFn=4, DFd=8, p=0.0674. WT mean=1.585; RAGE KO mean=0.9519; difference between means=0.6332; SE=0.08593; 95% CI=0.3946 to 0.8717. Geisser-Greenhouse's epsilon=0.6201.

C1qa: Two-way repeated measures ANOVA; genotype (WT vs RAGE KO) x HMGB1 concentration (0, 100, 1000 ng/ml)=11.28% of total variation, F=9.903, DFn=2, DFd=8, p=0.0069; HMGB1 concentration=56.94 % of total variation, F=49.99, DFn=1.231, DFd=4.925, p=0.0008; genotype =19.50% of total variation, F=10.11, DFn=1, DFd=4, p=0.0335; subject (n=3 per genotype; microglia cultured from 3 independent litters per genotype)=0.7.716% of total variation, F=3.387, DFn=4, DFd=8, p=0.0668. WT mean=0.1776; RAGE KO mean=0.05184; difference between means=0.1258; SE=0.03955; 95% CI=0.01595 to 0.2356.

Geisser-Greenhouse's epsilon=0.6157.

Ifnb: Two-way repeated measures ANOVA; genotype (WT vs RAGE KO) x HMGB1 concentration (0, 100, 1000 ng/ml)=5.379% of total variation, F=25.24, DFn=2, DFd=8, p=0.0004; HMGB1 concentration=83.15% of total variation, F=390.1, DFn=1.066, DFd=4.263, p<0.0001; genotype =9.840% of total variation, F=50.25, DFn=1, DFd=4, p=0.0021; subject (n=3 per genotype; microglia cultured from 3 independent litters per genotype)=0.7833% of total variation, F=1.838, DFn=4, DFd=8, p=0.2151. WT mean=0.9212; RAGE KO mean=0.4469; difference between means=0.4743; SE=0.06691; 95% CI=0.2885 to 0.6601. Geisser-Greenhouse's epsilon=0.5329.

Irf7: Two-way repeated measures ANOVA; genotype (WT vs RAGE KO) x HMGB1 concentration (0, 100, 1000 ng/ml)=6.295% of total variation, F=5.256, DFn=2, DFd=8, p=0.0349; HMGB1 concentration=66.05% of total variation, F=39.06, DFn=1.096, DFd=4.384, p=0.0023; genotype =12.00% of total variation, F=7.625, DFn=1, DFd=4, p=0.0508; subject (n=3 per genotype; microglia cultured from 3 independent litters per genotype)=6.295% of total variation, F=1.861, DFn=4, DFd=8, p=0.2108. WT mean=0.6971; RAGE KO mean=0.2295; difference between means=0.4676; SE=0.1693; 95% CI=-0.002545 to 0.9377. Geisser-Greenhouse's epsilon=0.5480.

Mx1: Two-way repeated measures ANOVA; genotype (WT vs RAGE KO) x HMGB1 concentration (0, 100, 1000 ng/ml)=6.282% of total variation, F=3.470, DFn=2, DFd=8, p=0.0822; HMGB1 concentration=72.94% of total variation, F=40.29, DFn=1.147, DFd=4.588, p=0.0018; genotype =8.310% of total variation, F=6.360, DFn=1, DFd=4, p=0.0652; subject (n=3 per genotype; microglia cultured from 3 independent litters per genotype)=5.226% of total variation, F=1.443, DFn=4, DFd=8, p=0.3046. WT mean=0.9991; RAGE KO mean=0.4258;

difference between means=0.5733; SE=0.2273; 95% CI=-0.05786 to 1.204. Geisser-Greenhouse's epsilon=0.5735.

C) Kruskal-Wallis statistic=78.89; p<0.0001. Mean rank mDNRAb$^-$=106.2, n=87; mDNRAb$^+$=215.1, n=77; mean rank difference=-109.0, z=8.801, p<0.0001. Mean rank RAGE KO mDNRAb$^-$=166.7, n=74; RAGE KO mDNRAb$^+$=168.7, n=86; mean rank difference=-1.986, z=0.1583, p=0.8742.

D) Linear mixed model analysis of estimated dendrite lengths; WT mDNRAb$^-$ mean=75.3, 95% CI= 67.7-82.8; WT mDNRAb$^+$ mean=54.0, 95% CI=46.1-61.9. Treatment x condition interaction effect B=29.107, 95% CI=13.563-44.651, p<0.001. Pairwise comparison of WT mDNRAb$^-$ vs. WT mDNRAb$^+$; estimated difference=-21.27, 95% CI= -35.6 to -6.91, p=0.0022.

E) Linear mixed model analysis of estimated dendrite lengths; RAGE KO mDNRAb$^-$ mean=78.5, 95% CI=70.7-94.1; RAGE KO mDNRAb$^+$ mean=86.3, 95% CI=78.6-94.1. Treatment x condition interaction effect B=29.107, 95% CI=13.563-44.651, p<0.001. Pairwise comparison of RAGE KO mDNRAb$^-$ vs. RAGE KO mDNRAb$^+$; estimated difference=7.84, 95% CI= -6.6 to 22.28, p=0.6192.

Figure 3.

A) Mann-Whitney U=10; p=0.0207. Median of DNRAb$^-$=1.081, n=8, sum of ranks=90; median of DNRAb$^+$=0.4826, n=8, sum of ranks=46. Difference between medians: -0.5981 (actual); -0.6209 (Hodges-Lehmann).

B) Mann-Whitney U=0; p=0.0286. Median of DNRAb$^+$ captopril=2.715, n=4, sum of ranks=26; median of DNRAb$^+$ enalapril=1.470, n=4, sum of ranks=10. Difference between medians: -1.245 (actual); -1.188 (Hodges-Lehmann).

D) Kruskal-Wallis statistic=20.08; p=0.0002. Mean rank WT DNRAb$^+$ saline=305.8, n=71; WT DNRAb$^+$ captopril=220.0, n=159. Uncorrected Dunn's test WT DNRAb$^+$ saline vs. WT DNRAb$^+$ captopril mean rank difference=85.75, z=4.282, p<0.0001. Mean rank LAIR-1 cKO DNRAb$^+$ saline=269.1, n=92; LAIR-1 cKO DNRAb$^+$ captopril=250.3, n=181. Uncorrected Dunn's test LAIR-1 cKO DNRAb$^+$ saline vs. LAIR-1 cKO DNRAb$^+$ captopril mean rank difference=18.82, z=1.048, p=0.2948.

E) Linear mixed model analysis of estimated dendrite lengths; LAIR-1 cKO DNRAb$^+$ captopril mean=27.5, SE=3.04, df=10, 95% CI=20.7-34.2; LAIR-1 cKO DNRAb$^+$ saline mean=24.4, SE=3.03, df=7, 95% CI=17.2-31.5; LAIR-1 cKO DNRAb$^-$ captopril mean=48.4, SE=3.68, df=7, 95% CI=39.7-57.1; LAIR-1 cKO DNRAb$^-$ saline mean=49.1, SE=3.03, df=7, 95% CI=42.0-56.3. Pairwise comparison of LAIR-1 cKO DNRAb$^+$ captopril vs. LAIR-1 cKO DNRAb$^+$ saline estimated difference=3.118, SE=4.29, df=7, t=0.726, p=0.8837. Pairwise comparison of LAIR-1 cKO DNRAb$^-$ captopril vs. LAIR-1 cKO DNRAb$^-$ saline estimated difference=-0.707, SE=4.76, df=7, t=-0.149, p=0.9987. Group effect=8.64988, SE=2.014263, df=9, t=4.294316, icc=0.135, p=0.0020.

Figure 4.

B) See Supplementary Tables 2 and 3.

E) See Supplementary Tables 4 and 5.

Supplementary Figure 1.

A) Linear mixed model analysis of estimated dendrite lengths; DNRAb$^+$ mean=25.1, SD=16.7; DNRAb$^-$ mean=31.1, SD=22.5. Pairwise comparison of DNRAb$^+$ vs. DNRAb$^-$ estimated difference=5.845406, SE=1.858640, df=7, t=3.144991, icc=0,213, p=0.0163.

B) Mann-Whitney U=30; p<0.0001. Median of 12 m.o. DNRAb$^-$=1.397, n=15, sum of ranks=360; median of 12 m.o. DNRAb$^+$ =1.039, n=18, sum of ranks=201. Difference between medians: -0.3575 (actual); -0.4144 (Hodges-Lehmann).

D) Mann-Whitney U=2214; p<0.0001. Median of 12 m.o. DNRAb$^-$=2.000, n=110, sum of ranks=8319; median of 12 m.o. DNRAb$^+$ =4.000, n=169, sum of ranks=30741. Difference between medians: 2.000 (actual); 1.000 (Hodges-Lehmann).

Supplementary Figure 2.

A) Kruskal-Wallis statistic=135.9; p<0.0001. Mean rank WT mDNRAb$^-$=258.5, n=72; WT mDNRAb$^+$=198.5, n=72; RAGE KO mDNRAb$^-$=159.1, n=96; RAGE KO mDNRAb$^+$=87.85, n=96. Uncorrected Dunn's test WT mDNRAb vs. WT mDNRAb$^+$ mean rank difference=59.94, z=3.705, p=0.0002; RAGE KO mDNRAb$^-$ vs. RAGE KO DNRAb$^+$ mean rank difference=71.28, z=5.087, p<0.0001.

B) Mann-Whitney U=355; p=0.0394. Median of LAIR-1 cKO DNRAb$^-$=59.00, n=29, sum of ranks=1095; median of LAIR-1 cKO DNRAb$^+$ =54.33, n=35, sum of ranks=985. Difference between medians: -4.670 (actual); -4.660 (Hodges-Lehmann).

Supplementary Figure 3.

See Supplementary Table 1.

Supplementary Figure 4.

A) Mann-Whitney U=6488458; p<0.0001. Median score of Homeostatic cluster=1.465, n=15285, sum of ranks=123311713; median score of Ms4a7+ cluster=1.983, n=2515, sum of ranks=35117188. Difference between medians: 0.5180 (actual); 0.5281 (Hodges-Lehmann).

B) Mann-Whitney U=1935571; p<0.0001. Median score of Homeostatic cluster=2.637, n=15285, sum of ranks=153329460; median score of Ms4a7+ cluster=0.9271, n=2515, sum of ranks=5099441. Difference between medians: -1.710 (actual); -1.697 (Hodges-Lehmann).

C) Mann-Whitney U=2280180; p<0.0001. Median score of Homeostatic cluster=-0.2318, n=15285, sum of ranks=119103435; median score of Ms4a7+ cluster=0.9713, n=2515, sum of ranks=39325466. Difference between medians: 1.203 (actual); 1.178 (Hodges-Lehmann).

D) Mann-Whitney U=6111440; p<0.0001. Median score of Homeostatic cluster=-0.4109, n=15285, sum of ranks=122934695; median score of Ms4a7+ cluster=2.182, n=2515, sum of ranks=35494206. Difference between medians: 2.593 (actual); 2.612 (Hodges-Lehmann).
